# Valorization of Lignin via Oxidative Depolymerization with Hydrogen Peroxide: Towards Carboxyl-Rich Oligomeric Lignin Fragments

**DOI:** 10.3390/molecules25112717

**Published:** 2020-06-11

**Authors:** Ulrike Junghans, Justin J. Bernhardt, Ronja Wollnik, Dominik Triebert, Gerd Unkelbach, Daniela Pufky-Heinrich

**Affiliations:** Fraunhofer Center for Chemical-Biotechnological Processes CBP, Am Haupttor, Bau 1251, 06237 Leuna, Germany; justin.bernhardt@igb.fraunhofer.de (J.J.B.); ronja.wollnik@yahoo.com (R.W.); dominik-triebert@web.de (D.T.); gerd.unkelbach@cbp.fraunhofer.de (G.U.); d.pufky@gmx.de (D.P.-H.)

**Keywords:** Kraft lignin, oxidative depolymerization, hydrogen peroxide, solvent fractionation, oligomeric lignin fragments

## Abstract

The extraction and characterization of defined and carboxyl-rich oligomeric lignin fragments with narrow molecular weight distribution is presented herein. With regard to the well-known pulp bleaching process, oxidative lignin depolymerization was investigated using hydrogen peroxide in an aqueous alkaline solution (i.e., at T = 318 K, t = 1 h) and subsequent selective fractionation with a 10/90 (*v*/*v*) acetone/water mixture. While the weight average molecular weight (M_W_) of lignin in comparison to the starting material was reduced by 82% after oxidation (T = 318 K, t = 1 h, c_lignin_ = 40 g L^−1^, c_H2O2_ = 80 g L^−1^, c_NaOH_ = 2 mol L^−1^) and subsequent solvent fractionation (T = 298 K, t = 18 h, c_cleavage product_ = 20 g L^−1^), the carboxyl group (–COOH) content increased from 1.29 mmol g^−1^ up to 2.66 mmol g^−1^. Finally, the successful scale-up of this whole process to 3 L scale led to gram amounts (14% yield) of oligomeric lignin fragments with a M_W_ of 1607 g mol^−1^, a number average molecular weight (M_N_) of 646 g mol^−1^, a narrow polydispersity index of 3.0, and a high –COOH content of 2.96 mmol g^−1^. Application of these oligomeric lignin fragments in epoxy resins or as adsorbents is conceivable without further functionalization.

## 1. Introduction

After cellulose, lignin is the second most abundant biopolymer on earth and the only highly available renewable source for aromatic hydrocarbons [1]. Yearly, 50 Mt lignin worldwide is generated from lignocellulosic biomass as a low-value byproduct in pulp and paper mills from the Kraft process [2,3,4]. Currently, this so-called Kraft lignin (KL) is mainly (98%) burned to generate heat and power for the pulping process and for the supply of renewable energy to the grid. Only 2% is used commercially as a specialty product [1,5]. However, due to an existing energy surplus, a higher percentage of the KL could be valorized without compromising the energy balance of the mills [6]. In addition, Hu et al. [2] stated that a future lignin biorefinery can only be realized if a stable and cheap feed supply can be realized, e.g., by upgrading a Kraft mill.

Nevertheless, the direct valorization of lignin waste remains a challenge because of its limited reactivity, caused by its large molecular weight and highly branched structure [7]. In addition, the lignin polymer is highly heterogeneous, as its macromolecular structure varies depending on its botanical origin, due to a wide variety of strong inter-unit linkages possible within the polymer [8,9]. The inherent heterogeneity of this biopolymer is further increased during the pulping process. However, the synthesis of high-value-added materials usually requires a narrow molecular weight distribution, as well as functionality and reactivity [10,11,12]. These three aims can be achieved via the depolymerization of lignin to smaller oligomeric hydrocarbons through application of thermochemical processes [13]. Essential processes, which have already been extensively discussed in several reviews, include gasification [14], pyrolysis [1], hydrogenolysis [15], hydrolysis [16], and chemical oxidation [5,17,18], yielding distinct products for various applications, e.g., as fuels [19], fuel additives [20], antifungal components [21], or for the preparation of polyurethane resins [22]. However, some of these processes require high temperatures and pressures and result in poorly functionalized aromatics or undesired byproducts [23,24,25].

To avoid these barriers, an alternative approach using mild reaction conditions is needed to retain or even further increase reactive functionalities, e.g., by incorporation of carboxylic groups into the molecule. One approach is the base-catalyzed depolymerization of lignin via addition of the environmentally friendly oxidant hydrogen peroxide (H_2_O_2_). Within pulp and paper production, H_2_O_2_ is applied for pulp bleaching to remove residual lignin from cellulose [26,27]. Hence, an oxidation process developed on the basis of pulp bleaching could be easily implemented in the process chains of paper mills. Recently, Ho et al. [28] reviewed the potential usage of alkaline hydrogen peroxide in lignocellulosic biomass pretreatment and valorization. However, the number of studies dealing with the oxidative depolymerization of isolated lignins with H_2_O_2_ over different catalysts is still limited [5,28]. For instance, softwood KL depolymerization with H_2_O_2_ was investigated by Napoly et al. [24] and He et al. [23]. Alternative approaches for the catalytic oxidation of lignin were recently reviewed by Liu et al. [29].

Thus, the present work was devoted to the extraction of carboxyl-rich oligomeric lignin fragments from softwood KL with a narrow molecular weight distribution. For the extraction of lignin oligomeric fragments, the oxidative depolymerization of softwood Kraft lignin with H_2_O_2_ in an aqueous sodium hydroxide solution (NaOH) was studied at temperatures up to 353 K, lignin concentrations up to 60 g L^−1^, H_2_O_2_ concentrations up to 120 g L^−1^, and NaOH concentrations up to 2 mol L^−1^. Note that these investigations were carried out in the absence of a catalyst. Simultaneously, structural changes of the depolymerized Kraft lignin accompanying the oxidation reaction were investigated. As shown by He et al. [23] for KL, lignin oxidation with H_2_O_2_ can introduce carboxyl groups into the lignin structure. Hence, an increasing carboxyl group content was expected for the oxidized Kraft lignin (OKL). In case of a high carboxyl group content within the OKL structure, it would be conceivable to directly apply the OKL, e.g., in low-temperature epoxy resin synthesis, without further functionalization, since depolymerized lignin post-functionalized with carboxyl groups has already been successfully used as a curing agent for epoxy resins [30,31]. Furthermore, application of OKL as an adsorbent for heavy metal ion removal from aqueous media would be conceivable, as the performance of such lignin-based adsorbents has been shown to be strong depending on the content of carboxyl groups [32,33]. In addition, the influence of a catalyst as well as the use of stabilized and non-stabilized H_2_O_2_ during oxidation on the molecular weight distribution and the yield of the OKL was examined.

Moreover, solvent fractionation of the solid OKL using different water/acetone ratios to achieve oligomeric lignin fragments with narrow molecular weight distribution (polydispersity index (PDI) ≤ 3) was investigated. Kraft lignin fractionation by multistage fractionation of distinct fractions from native lignin with reduced molecular weight and a low degree of polydispersity using organic solvents has already been reported [34,35,36,37]. Boeriu et al. [38] investigated the solvent fractionation of five technical lignins via selective fractionation in green solvents such as acetone/water mixtures or ethyl acetate. They obtained lower molecular weight lignin fractions with low polydispersity by fractionation with ethyl acetate and acetone/water mixtures containing 30% or 50% acetone. A different approach was reported by Liang and Wan [39], who investigated a sequential methanol fractionation followed by hydrogenolytic depolymerization, also in the presence of H_2_O_2_, using switchgrass-based lignin-rich residues from cellulosic ethanol production. However, to our knowledge, fractionation of OKL via oxidation with H_2_O_2_ has not yet been investigated. Finally, scale-up to generate larger samples of oligomeric lignin fractions with a narrow molecular weight distribution was studied at 3 L scale. Investigating the scaling of the laboratory processes is essential to validate possible future implementation at pulp and paper mills.

## 2. Results and Discussion

The feasibility of mild Kraft lignin oxidation with H_2_O_2_ in the presence and the absence of a catalyst was demonstrated. Parametric studies were performed in order to evaluate the influence of the process parameters (temperature, time, H_2_O_2_ concentration, NaOH concentration, and lignin concentration) on the product specifications of the lignin oligomeric fragments. The molecular weight distribution, functionality, O/C atomic ratio, structural changes of the products compared to the non-oxidized lignin, and the isolated yields of these products are discussed in detail. Additional molecular weight reduction of the lignin oligomeric fragments obtained via oxidative depolymerization with H_2_O_2_ was demonstrated by selective fractionation using acetone/water mixtures. Finally, the whole process chain (oxidation and solvent fractionation) was mapped to confirm the technology’s readiness for an industrial environment.

### 2.1. Oxidative Depolymerization of Kraft Lignin at a Laboratory Scale

#### 2.1.1. Influence of Reaction Parameters on Kraft Lignin Structure

At strong alkaline conditions (pH in the range of its pKa = 11.6 at 298 K [40]), H_2_O_2_ decomposes to water, oxygen, and an extremely strong nucleophilic hydroperoxide anion [41]. As summarized by He et al. [23], this anion reacts with lignin, causing cleavage at lignin side-chains or even benzene ring opening. Besides increasing the number of carboxyl, carbonyl, hydroxyl functions, and chromophore groups, these reactions decrease the average molecular weight of lignin. A detailed explanation of the possible reaction mechanisms of peroxides with lignin is given by Kadla et al. [42]. An overall oxidation scheme for the oxidation of softwood KL can be seen in Reference [23].

**Figure 1:**
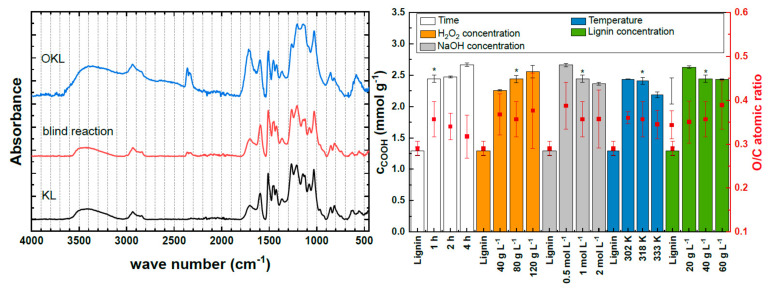
Attenuated total reflection infrared spectroscopy (ATR-IR) spectra of kraft lignin (KL), of lignin after the blind reaction, which was carried out in the absence of H_2_O_2_ and of oxidized Kraft lignin (OKL) (**left**), as well as carboxyl group content (c_COOH_) and O/C atomic ratio of KL and OKL in dependence of reaction parameters (**right**). The reference reaction is marked by an asterisk (reaction conditions: c_lignin_ = 40 g L^−1^, c_H2O2_ = 80 g L^−1^, c_NaOH_ = 1 mol L^−1^, T = 318 K, t = 1 h).

Structural changes within the lignin structure caused by the oxidation reaction were investigated by attenuated total reflection infrared spectroscopy (ATR-IR) analysis. Figure 1 (left) shows the ATR-IR spectra of KL before oxidation, and of OKL as well as of KL treated under reference reaction conditions in the absence of H_2_O_2_ (blind reaction). The specific bands were assigned according to Faix [43] and Boeriu et al. [44].

For the blind reaction, no structural changes within the lignin structure were observed, indicating that no oxidation reaction took place. However, when peroxide was added to the reaction solution, major changes in the ATR-IR spectrum of the OKL compared to the KL were observed. The OKL spectrum showed a broadening of the hydroxyl stretching band at 3600–3100 cm^−1^, corresponding to phenolic and aliphatic hydroxyl groups (–OH). A weak band at 2640 cm^−1^ indicated the formation of carboxylic acids (3200–2500 cm^−1^ [45]) during oxidation. Additionally, the phenolic –OH reacted during oxidation of KL, which was indicated by a decrease in intensity of the band at 1360 cm^−1^ in the OKL spectrum. This change was also observed by He et al. [23]. According to Kozhenikov et al. [46], another indication of successful oxidation of KL with H_2_O_2_ is given by an increasing intensity of the weak band at 1700 cm^−1^ in the OKL, which is associated with carbonyl/carboxyl stretch vibrations. However, in contrast to He et al. [23], under the applied reaction conditions in this work, aromatic cleavage was not observed, as no change of intensity of the aromatic skeleton vibrations at 1600 cm^−1^, 1500 cm^−1^, and 1417 cm^−1^ was observed for OKL compared to KL. Below 1400 cm^−1^, the bands within the spectra overlapped as different vibration modes contributed to the distinct bands. However, the strong band ranging from 1269–1022 cm^−1^ was assigned to the guaiacyl unit present in the softwood KL structure [44]. Additionally, and in agreement with He et al. [23], the relative intensity of the C=O stretching band at 1226 cm^−1^, which shifted within the spectrum to 1206 cm^−1^, increased within the OKL spectrum. This further indicated the formation of carboxyl groups (−COOH).

The possible formation of −COOH during oxidation of KL was confirmed by comparison of the amount of −COOH in KL and OKL (see Figure 1, right), measured via potentiometric analysis. Overall, the amount of −COOH was doubled in OKL from 1.29 mmol g^−1^ up to 2.66 mmol g^−1^. However, the −COOH content was not significantly influenced by variation of the reaction conditions, where a maximum difference of 0.48 mmol g^−1^ ranging from 2.18 mmol g^−1^ (at 333 K) to 2.66 mmol g^−1^ (at 4 h reaction time) was observed. Nevertheless, compared to He et al. [23], a 2.6-fold increase in −COOH content was observed. Simultaneously, and in agreement with the ATR-IR spectra shown in Figure 1 (left), an overall decrease in phenolic hydroxyl group (–OH) content was determined by potentiometric titration from 1.42 mmol g^−1^ in KL up to 0.83 mmol g^−1^ in OKL.

The elemental analysis further emphasized the successful oxidation of KL (see Figure 1 (right)). The O/C atomic ratio increased from 0.29 up to 0.39 after oxidation. This can probably be attributed to the increasing −COOH content within the OKL structure, as the overall trend of the O/C ratio in dependence of the reaction conditions followed the overall trend of the −COOH content.

#### 2.1.2. Influence of Reaction Parameters on Molecular Weight of Kraft Lignin

**Figure 2:**
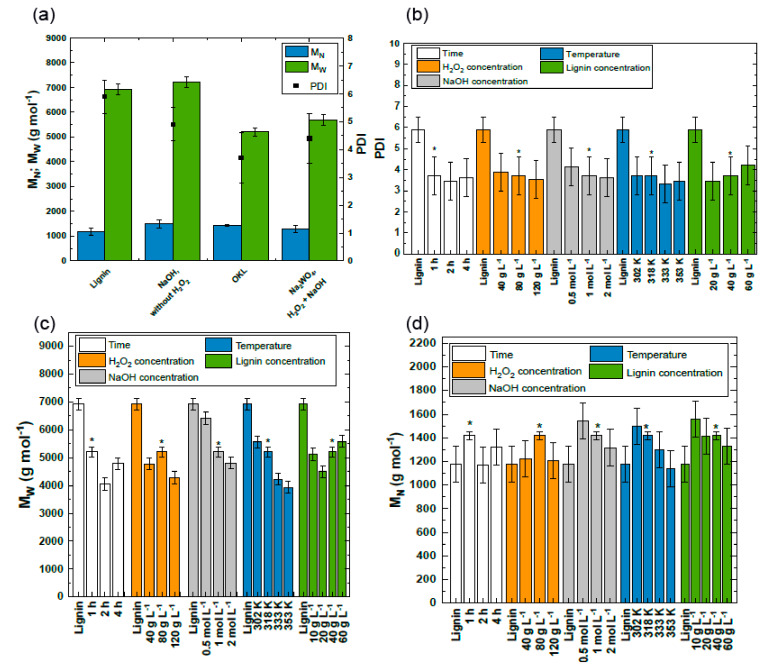
Weight average molecular weight (M_W_), number average molecular weight (M_N_), and polydispersity index (PDI) of lignin, lignin after the blind reaction, OKL, and OKL after application of Na_2_WO_4_ as a catalyst (**a**), as well as PDI (**b**), M_W_, and M_N_ of OKL ((**c**) and (**d**), respectively) in dependence on reaction parameters (reaction conditions: c_lignin_ = 40 g L^−1^, c_H2O2_ = 80 g L^−1^, c_NaOH_ = 1 mol L^−1^, T = 318 K, t = 1 h). In addition, the reference reaction is marked by an asterisk in (**b**–**d**).

As stated above, the oxidation of KL with H_2_O_2_ should lead to OKL, which exhibits a lower molecular mass and narrower polydispersity compared to KL. As can be seen in Figure 2a, no oxidation reaction occurred in the absence of H_2_O_2_ (blind reaction), as the molar mass remained constant within the measuring accuracy. This observation was in agreement with the above-discussed ATR-IR spectrum of the KL after reaction without H_2_O_2_. However, in accordance with the structural change (see Figure 1, left) in OKL under reference reaction conditions, the weight average molecular weight (M_W_) was reduced by 24.8%. Simultaneously, a 1.6-fold decrease of the PDI was observed, caused by a decrease in heterogeneity within the lignin macromolecule through the formation of more homogeneously distributed oligomeric depolymerization products (see Figure 2a,b). Interestingly, and in contrast to previous expectations, in the presence of the catalyst Na_2_WO_4_, a similar molar mass and PDI of OKL within the measuring accuracy was reached (see Figure 2a). As the catalyst showed no further influence on the oxidative depolymerization of KL, the investigated reaction was assumed to be entirely base-catalyzed by NaOH.

However, it was not assumed that β–O–4 ether linkages within the lignin molecule were cleaved. This would lead to a decrease in the number average molecular weight (M_N_) and M_W_ within OKL compared to KL, as observed by Rößiger et al. [13] for the base-catalyzed depolymerization of lignin at pressures and temperatures above 250 bar and 270 °C, respectively, and by He et al. [23] for the base-catalyzed depolymerization of lignin with H_2_O_2_ at temperatures above 318 K. Interestingly, Figure 2c,d shows decreasing M_W_, but within the measuring accuracy a constant or only slightly increasing M_N_. This led to the conclusion that the molecular weight change with respect to the total number of molecules within the oligomeric lignin fraction remained constant. Probably, under the investigated reaction conditions, only a slight structural change in the lignin oligomer was observed, as the oxidation reaction occurs primarily at the lignin side-chains by cleaving lower molecular weight compounds present in lignin. The removal of these low-molecular-weight compounds during the purification of the OKL (see Section 2.2) led to the observed constant or slightly increased M_N_. Nevertheless, throughout the reaction parameter variation experiments, the M_W_ and M_N_ of OKL generally showed the same trend within the measuring accuracy.

As can be seen from Figure 2c, upon increasing the NaOH concentration as well as the reaction temperature, an up to 6.1-fold decrease in weight average molecular weight (M_W_ = 1139 g mol^−1^ at 353 K) was observed. Increasing both parameters promoted the formation of the hydroperoxide anion, leading to a stronger KL depolymerization. However, above 1 mol L^−1^ NaOH or 333 K, M_W_ was not significantly decreased anymore due to a highly accelerated decomposition of H_2_O_2_, which decreased the probability for lignin depolymerization [23,47]. In addition, upon increasing H_2_O_2_ concentration or decreasing lignin concentration an up to 1.6-fold decrease in M_W_ was observed, as an increasing concentration of hydroperoxide anions could depolymerize a larger amount of lignin. Surprisingly, at 10 g L^−1^ KL, a higher M_W_ was observed compared to 20 g L^−1^ KL (5125 g mol^−1^ vs. 4492 g mol^−1^). This can be attributed to the fact that at 10 g L^−1^ KL, a 2.4-fold excess of H_2_O_2_ compared to the applied KL mass was present in the reaction solution. Hence, the oxidation was probably very fast, resulting in the recombination of oligomeric fragments or monomers with the bulk lignin, or the H_2_O_2_ excess led to a pronounced unproductive decomposition of H_2_O_2_, resulting in lower lignin depolymerization.

Previously, similar results were obtained by He et al. [23]. However, they did not observe an influence of reaction time on the oxidation reaction at temperatures above 333 K. Figure 2c shows that at 318 K, M_W_ decreased after 2 h of reaction time to 4055 g mol^-1^, and increased again to 4784 g mol^−1^ after 4 h of reaction time. This can probably be attributed to the fact that the oxidation reaction was completed before 4 h of reaction time, resulting in the above-mentioned recombination of fragments with the bulk lignin. Nevertheless, by variation of the reaction parameters, oligomeric KL fragments with a M_W_ between 6411 g mol^−1^ and 3937 g mol^−1^, a M_N_ between 1545 g mol^−1^ and 1170 g mol^−1^, and a PDI between 4.1 and 3.3 were obtained (see Figure 2b–d). Note that a combination of these parameters did not result in any further reduction of the molar mass of the obtained OKL and yields between 58% and 95% were obtained. Additional 18% to 4% yields were obtained as liquid product named oil-OKL, exhibiting a M_W_ between 810 g mol^−1^ and 526 g mol^−1^, a M_N_ between 526 g mol^−1^ and 399 g mol^−1^, and a PDI between 1.9 and 1.5. Interestingly, the obtained average molecular weight of the oil-OKL was independent of the applied reaction parameters. Monomers present within the oil-OKL were apocynin and vanillin. The remaining 2%–24% needed to close the mass balance can probably be accounted for by small molecules like methanol, formiate, or formic acid, which were not analyzed in this work, or to loss of OKL during separation of the OKL from the liquid phase by centrifugation. During acidification of the reaction solution, a small part of the OKL was suspended in the reaction solution and was not recovered during our studies. In addition, as for the base-catalyzed depolymerization, gaseous degradation products (e.g., CO_2_) of KL can probably be formed as well [48]. However, no analysis of the gas phase was carried out, as these products were outside the scope of this study.

### 2.2. OKL Solvent Fractionation at a Laboratory Scale

**Figure 3:**
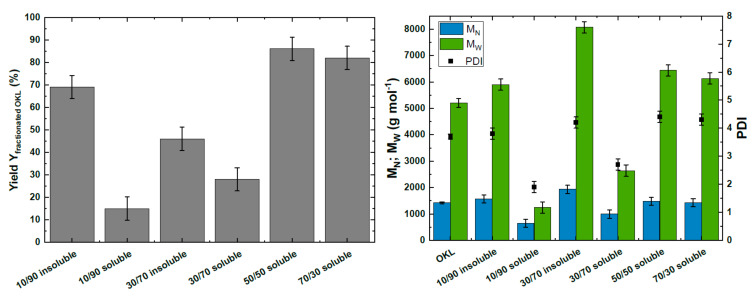
Yield Y of fractionated OKL (**left**) and M_W_, M_N_, and PDI of OKL and of fractionated OKL (**right**) after solvent fractionation in 10/90, 30/70, 50/50, and 70/30 acetone/water (*v*/*v*) mixtures.

In order to yield defined homogeneous oligomeric fragments from lignin for polymer applications, the molecular weight distribution described above and thus the polydispersity of the OKL needed to be further narrowed. This was successfully achieved by fractionation of OKL with acetone/water mixtures. Figure 3 (left) shows the yield of the corresponding OKL after fractionation. Using 50 vol. % and 70 vol. % acetone, the OKL was nearly completely dissolved in the acetone/water mixture. The respective insoluble OKL fractions could not be recovered from the filter. Nevertheless, both soluble OKL fractions were obtained with yields greater than 80%. Below 50 vol. % acetone within the aqueous phase, the solubility of the OKL decreased with increasing water content, resulting in both soluble and insoluble OKL fractions with cumulative yields (including yield of soluble and insoluble fraction) of 84% (10/90 (*v*/*v*) acetone/water) and 74% (30/70 (*v*/*v*) acetone/water, respectively). Note that the residue mass balance can be accounted for by losses occurring during recovery of the fractionated OKL.

Similar results for the solubility behavior were obtained by Boeriu et al. [38] for the solvent fractionation of virgin KL. The authors explained the solubility behavior of lignin in different solvent mixtures using the solubility parameter theory of Hildebrandt and Scott [49]. This theory states that an oligomer can be dissolved in a solvent that exhibits a similar solubility parameter [38,49]. By calculation of these parameters, Boeriu et al. [38] showed that KL exhibits a Gaussian solubility pattern, reaching a maximum solubility in mixtures with 50 vol. %–90 vol. % acetone. Accordingly, we assumed that the OKL would follow the same behavior. Hence, the yield of the soluble OKL fraction nearly doubled from 10 vol. % to 30 vol. % acetone, while a 1.5-fold decrease of the yield of the insoluble OKL fraction was observed. Interestingly, Boeriu et al. [38] observed a 5-fold increase for the soluble KL fraction at the same volume ratios of acetone (10 vol. % to 30 vol. %). This was probably caused by the higher heterogeneity of the KL oligomers compared to the OKL oligomers, resulting in broader polarity differences of the distinct molecules within KL compared to OKL.

The obtained molecular weight distribution of the OKL after fractionation with acetone/water mixtures can be seen in Figure 3 (right). It was observed that the lower the acetone concentration within the solvent mixture, the lower the molecular mass of the fractionated OKL that was obtained within the soluble fraction (see Figure 3, right). As expected, no fractionation was observed at 50 vol.% and 70 vol. % acetone. In contrast, soluble OKL fractions with 2-fold and 4.2-fold smaller M_W_ and narrow PDIs of 2.7 and 1.9 were obtained at 30 vol. % and 10 vol. % acetone, respectively. If an average molecular mass of 180 g mol^−1^ [23] per lignin unit is assumed, the soluble OKL contained oligomers consisting of approximately seven to ten monomer units. Interestingly, a 1.1- to 1.6-fold increase of M_W_ for the insoluble OKL fractions with a similar PDI to the non-fractionated OKL was observed at 10 vol. % and 30 vol. % acetone, respectively (see Figure 3, right). According to previous works [12,28,50], lignin aggregation can occur in solution, caused by π–π stacking resulting in a higher molecular weight. The results obtained here were in agreement with the observations made by Boeriu et al. [38] for the solvent fractionation of KL. The only exception was the absence of the lignin–carbohydrate complex formation within the soluble OKL fraction at 10 vol. % acetone. Overall, the oxidative depolymerization of KL with H_2_O_2_ followed by a 10/90 (*v*/*v*) acetone/water fractionation of the resulting OKL was successfully carried out, yielding oligomeric fragments with a narrow molecular weight distribution (M_W_ = 1241 ± 216 g mol^−1^, M_N_ = 645 ± 153 g mol^−1^, PDI = 1.9 ± 0.2).

### 2.3. Oxidative Depolymerization of Kraft Lignin and OKL Solvent Fractionation at a 3 L Scale

#### 2.3.1. Preliminary Studies on Application of Stabilized H_2_O_2_ at a Laboratory Scale

The above-discussed extraction route for oligomeric fragments with narrow molecular weight distribution can only add value to lignin use if the process can be scaled-up. From an economic point of view, KL oxidation should be carried out with stabilized H_2_O_2_. Hence, prior to the scale-up tests, its applicability for the oxidative depolymerization of KL was tested at a laboratory scale. The corresponding results are shown in Table 1.

Stabilized H_2_O_2_ was more efficiently used for the oxidation reaction than non-stabilized H_2_O_2_, as the obtained molecular weight of the corresponding OKL was smaller (1.7-fold for M_N_, 1.8-fold for M_W_) than for the OKL obtained with non-stabilized H_2_O_2_. The PDI remained constant for both oxidizing agents. In addition, both M_N_ and M_W_ decreased with stabilized H_2_O_2_, which was in contrast to the above-discussed increase of M_N_ for the OKL, but in agreement with the results obtained by He et al. [23]. As already stated above, decreasing M_N_ and M_W_ values indicate β–*O*–4-ether linkage cleavage [13,23]. However, as the ether cleavage generally occurs at higher temperatures and as the PDI of the OKL for both oxidizing agents remained the same, no β–*O*–4-ether linkage cleavage in the OKL was assumed. Hence, the lower molecular weight of the OKL with stabilized H_2_O_2_ was probably caused by a higher degradation degree at the side-chains of the lignin oligomer. Additionally, Kadla et al. [47] also observed a more efficient lignin degradation in the presence of a stabilizing agent. This suggests that the stabilizing agent (sodium pyrophosphate) within the H_2_O_2_ used also had a stabilizing effect on the lignin degradation products, preventing possible further condensation. Interestingly, for the OKL obtained with stabilized H_2_O_2_, a 1.5-fold smaller cumulative yield was gained compared to the yield of OKL obtained with non-stabilized H_2_O_2_. This observation contributed to the assumption of increased side-chain degradation leading to higher gas and colloidal particle formation, which could not be recovered during centrifugation. Hence, for the scaled-up process, the separation of the OKL from the aqueous solution was modified to filtration in order to achieve recovery of the colloidal molecules and to reduce yield losses.

#### 2.3.2. Transition of Laboratory-Scale Oxidation and Solvent Fractionation to 3 L Scale

The oxidation of KL with H_2_O_2_ at a 3 L scale was carried out with reproducible results. The corresponding M_N_, M_W_, and PDI values of the obtained OKL as well as the cumulative yield of the OKL are shown in Table 1. Due to application of filtration of the OKL from the aqueous solution, a high cumulative yield compared to the laboratory-scale experiment with non-stabilized H_2_O_2_ was obtained. As the colloidal molecules were successfully separated together with the residue solid OKL, a 1.3-fold higher PDI for the 3 L scale experiment was obtained compared to the laboratory-scale experiment. In addition, a 1.1-fold and a 1.5-fold decrease of M_N_ and M_W_, respectively, of the OKL compared to the KL was observed. However, the molecular weight of the obtained fraction was higher than for the laboratory experiment. This could be attributable to the method of H_2_O_2_ addition. At the 3 L scale, H_2_O_2_ was added dropwise to the reaction solution to prevent strong foam formation, while at the laboratory scale, the H_2_O_2_ was added all at once (see Section 2.2). Although the reaction was run for 1 h after full addition of the H_2_O_2_, the reaction had already begun, with the first drop of H_2_O_2_ resulting in the first depolymerization products (e.g., monomers), which could recombine with the bulk lignin during the following 1 h of reaction time. This assumption was supported by the lower oil-OKL yield (Y_oil_ = 7.6 ± 0.3%) obtained at the 3 L scale compared to the laboratory scale (Y_oil_ = 11 ± 3%). The remaining 13% yield for the 3 L scale can again be attributed to losses during separation and purification of the OKL, as well as gas/side product formation during the oxidation reaction.

**Figure 4:**
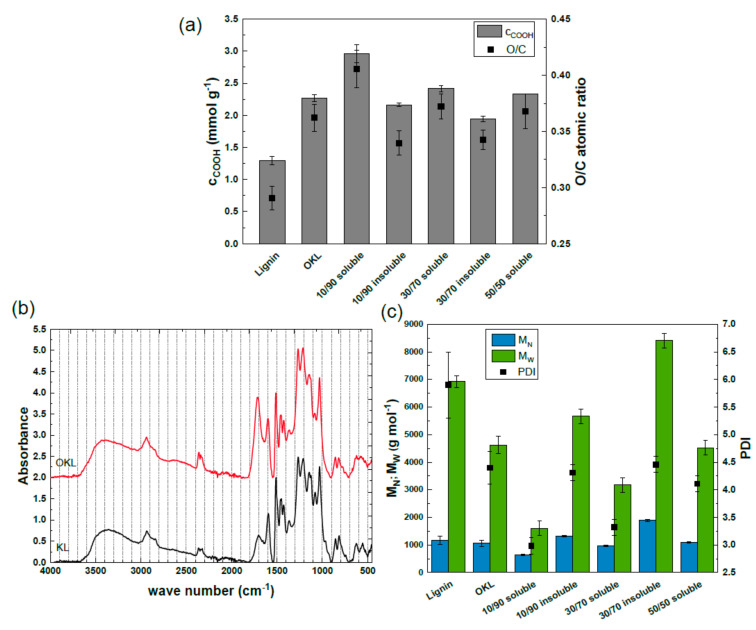
Carboxyl group content and O/C atomic ratio (**a**) of lignin (KL), OKL (reaction conditions: c_lignin_ = 40 g L^−1^, c_H2O2_ = 80 g L^−1^, c_NaOH_ = 2 mol L^−1^, T = 318 K, t = 1 h), and fractionated OKL; ATR-IR spectra of lignin (KL) and OKL (**b**) as well as M_W_, M_N_, and PDI (**c**) of lignin (KL), OKL, and fractionated OKL after oxidation of KL and solvent fractionation in 10/90, 30/70, 50/50 acetone/water (*v*/*v*) mixtures at a 3 L scale.

However, despite the observed deviation in molecular mass distribution of the OKL, the O/C atomic ratio as well as the −COOH content increased in the same range as for the laboratory scale, from 0.29 in KL to 0.36 in OKL and from 1.29 mmol g^−1^ in KL to 2.26 mmol g^−1^ in OKL under the investigated reaction conditions (2 mol L^−1^ NaOH, 318 K, 1 h; see Figure 4a). In addition, the ATR-IR spectra of the OKL obtained by oxidation of KL with H_2_O_2_ at the 3 L scale showed the same changes of band intensities as already discussed in Section 2.1.1 (see Figure 4b). Hence, successful oxidation was achieved at a 3 L scale.

In the following, the obtained OKL was solvent-fractionated according to the procedure developed at the laboratory scale to achieve oligomeric fragments with a narrow molecular weight distribution. Similarly to the laboratory scale, with 50 vol. % acetone, the OKL was completely dissolved in the solvent mixture. However, this fraction was obtained with a lower yield (58%) compared to the laboratory scale (above 80%), due to significant yield losses during recovery of the OKL. Hence, part of the OKL could not be recovered from the batch reactor. However, below 50 vol. % acetone, soluble and insoluble OKL fractions were obtained with cumulative yields above 97%.

The corresponding molecular weight distribution of the OKL after fractionation with acetone/water mixture is shown in Figure 4c. Similarly to the laboratory results, it was observed that the lower the acetone concentration in the solvent mixture, the lower the molecular mass of the fractionated OKL obtained within the soluble fraction. Compared to the OKL the soluble OKL fractions showed a 1.5-fold and 2.9-fold decrease of M_W_ and a narrow PDI of 3.3 and 3 at 30 vol. % and 10 vol. % acetone, respectively. Again, if an average molecular mass of 180 g mol^−1^ [23] per lignin unit is assumed (see Section 3.2), the soluble OKL contained oligomers consisting of approximately eight to 17 monomer units, which is up to 1.7-fold higher than on the laboratory scale. This was probably caused by a higher polarity of the OKL after oxidation with stabilized H_2_O_2_, resulting in a higher solubility of oligomers with higher molecular mass compared to the laboratory scale. Again, lignin aggregation was observed at 10 vol. % and 30 vol. % acetone for the insoluble OKL fractions. Both fractions showed a similar PDI to the non-fractionated OKL, but a 1.2-fold or 1.8-fold higher M_W_, respectively.

The −COOH content and O/C atomic ratio of the respective soluble and insoluble OKL fractions are shown in Figure 4a. Generally, the −COOH content of the soluble OKL fractions was higher than the −COOH content of the insoluble OKL fractions. The OKL fraction with the highest −COOH content of 3.0 mmol g^−1^ was the soluble fraction generated using 10 vol. % acetone. It even had a 1.3-fold higher −COOH content compared to the unfractionated OKL. The one with the lowest −COOH content (1.9 mmol g^−1^) was the agglomerated fraction generated using 30 vol. % acetone. Hence, the higher the –COOH content, the higher the polarity and the higher the hydrophilicity of the OKL fractions. Accordingly, the higher the –COOH content of the OKL fractions, the higher the solubility in acetone/water solutions with decreasing acetone content. In addition, as the OKL fraction was completely dissolved in a 50/50 (v/v) acetone/water mixture, no change in the molecular weight distribution, the −COOH content, or the O/C atomic ratio was observed (see Figure 4a,c).

In agreement with the observations made for the lignin oxidation in Section 2.1.1, the elemental analysis followed the same trend as the −COOH content (see Figure 4a). Similar observations concerning the polarity and solubility behavior of native lignin fractions in water/acetone mixtures were made by Boeriu et al. [38]. Overall, the scaled-up oxidative depolymerization of KL with H_2_O_2_ followed by 10/90 (v/v) acetone/water fractionation of the resulting OKL was successfully carried out, yielding oligomeric lignin fragments with a narrow molecular weight distribution (M_W_ = 1607 ± 264 g mol^−1^, M_N_ = 646 ± 28 g mol^−1^, PDI = 3.0 ± 0.1) and with high −COOH content (2.96 mmol g^−1^) at a multi-gram scale for further application tests. As outlined by Upton et al. [51] and demonstrated by Liu et al. [31] and Qin et al. [30], the incorporation of carboxyl groups into lignin facilitates its application as a hardener for epoxy resins without the need for a second commercial petroleum-based one, due to the higher reactivity of carboxyl groups compared to phenolic groups. Further, as shown by Jawerth et al. [52] and Gioia et al. [53], a PDI of OKL of 3.0 is suitable for thermosetting materials like epoxy resins, and the reduction of M_W_ results in an increase of solubility of OKL in various solvents (e.g., tetrahydrofuran), which have to be applied in epoxy resin synthesis. Therefore, application of OKL as hardener compound for epoxy resin synthesis would be conceivable without further functionalization. Due to their high −COOH content, the OKLs in this study could probably also be used directly as adsorbents for the separation of heavy metals from aqueous media [32,33], or, as investigated by He et al. [23], as anionic dispersants for clay suspensions. The authors observed that carboxyl-rich oligomeric lignin fragment addition to a clay suspension increased the zeta potential of the particles within this suspension. This impact of OKL on the zeta potential is also useful in binder applications (e.g., in feed pellets [54], as asphalt binder modifiers [55]). Hence, as the studied OKLs are charged molecules, they could even be conceivable low-sulfur-containing lignin alternatives in common lignosulphonate applications. Lignosulphonates are also used as binders and dispersants or inter alia as pore-forming agents in lead–acid batteries to improve service life, cold-start performance, and capacity [54,56]. In summary, in this study, the properties of the investigated Kraft lignin could be tuned by oxidation and subsequent solvent fractionation, probably allowing the use of these OKLs for a broad spectrum of applications, which may in the future result in commercially available lignin-containing products, if cost-effective production routes compared to conventional petroleum-based production routes can be developed.

## 3. Materials and Methods

### 3.1. Materials

Kraft lignin (Domtar BioChoice^TM^), which was precipitated from Kraft black liquor of pine wood using the LignoBoost^®^ process, was used in this work. Aqueous hydrogen peroxide (H_2_O_2_, 30% in water, AnalaR NORMAPUR^®^, Merck), stabilized aqueous H_2_O_2_ (30% in water, AnalaR NORMAPUR^®^, Merck, stabilizer: sodium pyrophosphate 0.02%), sodium hydroxide (≥ 99%, Merck), sulfuric acid (H_2_SO_4_, 25%, Carl Roth), sodium tungstate dihydrate (Na_2_WO_4_·2H_2_O, 99%, VWR), methyl isobutyl ketone (MIBK, 100%, GPC RECTAPUR^®^, VWR), sodium sulfate (Na_2_SO_4_, ≥ 99%, EMSURE^®^ Merck), and acetone (99%, VWR) were applied during lignin oxidation or fractionation experiments. Fluoranthene (100%, Supelco^®^) was applied in GC analysis. If not otherwise stated, all chemicals were used without further purification.

### 3.2. Oxidative Depolymerization of Kraft Lignin with Hydrogen Peroxide

The oxidation procedure was inspired by Napoly et al. [24]. Note, in contrast to Napoly et al. [24], the majority of the experiments were carried out without a catalyst and the oligomeric fragments were the focus of this investigation. In addition, the experiments of Napoly et al. [24] were exclusively laboratory-scale experiments.

At the laboratory scale, the experiments were carried out batch-wise in a two-neck round-bottom glass reactor (V = 1 L) equipped with a reflux condenser and a magnetic stirrer at 500 rpm. The scaled-up process was carried out in a 10 L glass reactor (Normag) equipped with a KPG stirrer at 105 rpm, a Huber Unichiller WC012, and a Huber Unistat 240-CC-NR. In a typical experiment, Kraft lignin (6 g–18 g; M_N_ = 1177 ± 153 g mol^−1^, M_W_ = 6920 ± 216 g mol^−1^) dissolved (10 g L^−1^–60 g L^−1^, with respect to aqueous NaOH solution) in 300 mL NaOH (0.5 mol L^−1^–2 mol L^−1^) was loaded into the reactor (conditions at scale-up: 120 g KL, 3 L 2 mol L^−1^ NaOH). After equilibrating the reaction mixture for 10 min at the reaction temperature (318 ± 1 K–353 K), aqueous H_2_O_2_ solution (12 g–36 g, 2 eq. with respect to the lignin mass, 40 g L^−1^–120 g L^−1^ with respect to aqueous NaOH solution, scale-up: 240 g stabilized H_2_O_2_) was added to start the reaction. For the scaled-up process, the aqueous H_2_O_2_ solution was added dropwise. The oxidation reaction was carried out for 1 to 4 h. Note, that for reaction temperatures above 318 K, the reaction was quenched in a water bath to 318 K after the specific reaction time. In one experiment, the oxidation reaction was carried out in the presence of 3.45 g (10.5 mmol) Na_2_WO_4_·2H_2_O as a catalyst, which was also applied by Napoly et al. [24].

The purification and separation of the reaction products was adapted from an in-house method from Rößiger et al. [13]. The complete reaction mixture was acidified to pH 1 ± 0.05 by addition of sulfuric acid. The precipitated light brown solid (OKL) was separated from an orange aqueous solution (containing low-molecular-weight depolymerization products and oil-OKL) either by centrifugation or by filtration. The centrifugation was conducted at 10,000 *g* at 293 K for 15 min. For salt removal, the solid precipitate was then suspended in 250 mL deionized water and centrifuged again under the same operation conditions. This procedure was repeated three more times. In case of filtration, the solution containing the precipitated OKL was first heated for 5 min at 338 K at 105 rpm, then cooled to 308 K and afterwards filtered using filter paper (Whatman grade 5, 2.5 µm, scale-up: Whatman grade 597, 4–7 µm). Note that the separation of the OKL in the scaled-up process was carried out solely by filtration.

The purified OKL from both the laboratory experiments and the scaled experiments was dried to a constant weight at 313 K under a vacuum.

The supernatant containing the low-molecular-weight phenolic lignin products and the supernatant from the first solid washing were combined and extracted three times with 100 mL MIBK each. The three extracts were combined and dried with anhydrous Na_2_SO_4_, which was filtered afterwards and washed with 25 mL MIBK. The MIBK was then removed from the combined extract by vacuum distillation. The obtained orange-brown oil-OKL was dried to a constant weight at 313 K under vacuum.

In case of the scaled-up process, 6 L supernatant was obtained. Aliquots of 3 L were extracted three times with 250 mL MIBK each. The MIBK was then removed from the obtained extract by vacuum distillation and reused in the next extraction experiment. Note that the recovered MIBK was constantly checked for impurities by gas chromatography analysis. At the laboratory scale, the obtained oil-OKL was dried to a constant weight at 313 K under vacuum.

### 3.3. Solvent Fractionation of Oligomeric Depolymerization Products

The fractionation procedure was adapted from Boeriu et al. [38], although they only fractionated native lignin. A measure of 2 g (scale-up: 40 g) lignin depolymerization product was suspended in 100 mL (scale-up: 2 L) of differently concentrated (90/10 (*v*/*v*), 70/30 (*v*/*v*), 50/50 (*v*/*v*), and 30/70 (*v*/*v*)) H_2_O/acetone solution. After stirring for 18 h at 375 rpm at room temperature, a beige suspension (at a composition of 90/10 (*v*/*v*), 70/30 (*v*/*v*)) to dark brown solution (at a composition of 50/50 (*v*/*v*) and 30/70 (*v*/*v*)) was obtained. The insoluble fractions were filtered and dried to a constant weight at 313 K and 20 mbar. The soluble lignin depolymerization product fraction was recovered by vacuum distillation after removal of acetone at 313 K and 150 mbar, followed by water removal at 333 K at 80 mbar. The obtained brown solid was dried to a constant weight at 313 K and 20 mbar.

### 3.4. Analytical Characterization of Kraft Lignin and Reaction Products

The yield of the obtained depolymerization products, solid (*Y*_s_) and oil (*Y*_oil_), was calculated using the following Equations (1) and (2).

*Y*_s_ = (*m*_s_ × (100% − *w*_water,s_ − *w*_ash,s_)) / (*m*_L_ × (100% − *w*_water,*L*_ − *w*_ash,L_) × 100% (1)

*Y*_oil_ = (*m*_oil_ × (100% − *w*_MIBK,oil_)) / (*m*_L_ × (100% − *w*_water,*L*_ − *w*_ash,L_) × 100%, (2)

where m_s_: mass of obtained solid; w_water,s_: residue water content of the solid; w_ash,s_: ash content of the solid; m_L_: mass of unreacted Kraft lignin; w_water,L_: residue water content of unreacted Kraft lignin; w_ash,L_: ash content of unreacted Kraft lignin; m_oil_: mass of obtained oil; w_MIBK,oil_: residual MIBK content within the oil.

Data on the yield of the depolymerization products were reproducible within ±5%. The determination of w_water,s_, w_water,L_, w_ash,s_, and w_ash,L_ was carried out according to a laboratory analytical procedure [57]. The w_MIBK,oil_ was determined by gas chromatography (GC-FID, Trace 1310, Thermo Scientific equipped with a flame ionization detector). Product separation with GC-FID was achieved on a Rtx^®^-1 capillary column (105 m length, 0.25 mm inner diameter, 0.5 µm film thickness, Restek). In addition, identification of the low-molecular-weight phenolic compounds within the oil was achieved via gas chromatography–mass spectrometry (GC-MS, 5970 VL MSD, Agilent Technologies). Product separation was achieved on a capillary column (HP-5MS (GC-MS), Agilent J&W, 30 m length, 0.25 mm inner diameter, 0.33 µm film thickness). Fluoranthene was used as an internal standard.

The molecular weights (number average molecular weights M_N_, weight average molecular weights M_W_) of the solid and the oil were determined by gel permeation chromatography (GPC, HPLC series 1260 Infinity II, Agilent Technologies) equipped with a series of two GPC columns (ABOA DMSO-Phil-P-250 and -350, AppliChrom, 0.3 m length 8 mm inner diameter) and a refractive index detector. The molecular weight was determined via the external standard method using dextrans with molecular weights between 180 g mol^−1^ to 9900 g mol^−1^ as standards. Data on molecular mass M_N_ and M_W_ could be determined with GPC within ±153 g mol^−1^ and ±216 g mol^−1^, respectively.

Attenuated total reflection infrared spectra (ATR-IR) of the solids were measured with an ALPHA spectrometer (Bruker) equipped with a single reflection diamond crystal. Scans were taken from 4000–400 cm^−1^, with a resolution of 4 cm^−1^, and 20 scans were averaged (software Bruker OPUS 7.5.18). The resulting spectra were background-subtracted, baseline-corrected, and normalized at 1500 cm^−1^. The determination of the free carboxylic acid and phenolic hydroxide functionalities of the solids was carried out by potentiometric titration with a 888 Titrando (ΩMetrohm) by application of a literature method [58], analyzing each sample twice. Elemental analysis (CHNS) of the solids and Kraft lignin was carried out via DIN EN 15104 at Deutsches Biomasseforschungszentrum (DBFZ), analyzing each sample three times. The oxygen content was calculated from the results of the elemental analysis, after correction of the elemental analysis results by the ash and residual water content using Equation (3).

*w*(O _wf, af_) = 1 − (*w*(C _wf, af_) + *w*(N _wf, af_) + *w*(S _wf, af_)) + *w*(H _wf, af_)), (3)

where _wf_: water free, _af_: ash free.

## 4. Conclusions

In the present work, the oxidative depolymerization of Kraft lignin with H_2_O_2_ in an aqueous alkaline solution followed by solvent fractionation (10/90 (*v*/*v*) water/acetone) of the oligomeric fragments was successfully demonstrated. This process yielded carboxyl-rich oligomeric lignin fragments with lower molecular weights and narrower molecular weight distribution compared to the starting material. The increase of functionality was indicated by ATR-IR analysis of the oligomeric fragments and confirmed by elemental analysis and quantification of carboxyl and phenolic groups with potentiometric titration, while the decrease in molecular weights and polydispersity was confirmed by gel permeation chromatography. Compared to the starting material, the weight average molecular weight (M_W_) of lignin was reduced by 82% after oxidative depolymerization (T = 318 K, t = 1 h, c_lignin_ = 40 g L^−1^, c_H2O2_ = 80 g L^−1^, c_NaOH_ = 2 mol L^−1^) followed by solvent fractionation (T = 298 K, t = 18 h, c_cleavage product_ = 20 g L^−1^) at the laboratory scale, while the carboxyl group (–COOH) content increased from 1.29 mmol g^−1^ up to 2.66 mmol g^−1^. Interestingly, the application of an oxidation catalyst (Na_2_WO_4_) had no influence on the depolymerization degree or molecular weight of the oligomeric fragments. Furthermore, stabilized H_2_O_2_ was shown to be a more efficient lignin degradation agent than non-stabilized H_2_O_2_. The successful translation of this process to a 3 L scale showed comparable results to the laboratory scale and yielded gram amounts of the highly functional and homogeneous oligomeric fragments. Here, the M_W_ was decreased by 77% (M_W_ = 1607 g mol^−1^) and the polydispersity index by 49% (PDI = 3.0) compared to the starting material after oxidation and solvent fractionation, while the carboxyl group content was increased from 1.29 mmol g^−1^ up to 2.96 mmol g^−1^. Due to these properties, the application of the oligomeric lignin fragments in a broad spectrum of polymer material applications, such as green hardener compounds in low-temperature epoxy resin synthesis or as green binding agents or dispersants, would be conceivable.

## Figures and Tables

**Table 1:** M_W_, M_N_, and PDI of KL and OKL after oxidation with non-stabilized and stabilized H_2_O_2_ at a laboratory scale and a 3 L scale, as well as respective OKL yields (reaction conditions: c_lignin_ = 40 g L^−1^, c_H2O2_ = 80 g L^−1^, c_NaOH_ = 2 mol L^−1^, T = 318 K, t = 1 h).

Sample/Treatment/Scale	M_N_ (g mol^−1^)	M_W_ (g mol^−1^)	PDI	Y (%)
Kraft lignin	1177 ± 153	6920 ± 216	5.9 ± 0.6	n.a.^a^
H_2_O_2lab scale_	1362 ± 62	5170 ± 365	3.7 ± 0.1	69 ± 9
H_2_O_2stabilized, lab scale_	801 ± 153	2802 ± 216	3.5 ± 0.6	47 ± 14
H_2_O_2stabilized, 3 L scale_	1058 ± 121	4625 ± 322	4.4 ± 0.3	73 ± 3

^a^ n.a.: not applicable.
